# Clinical Efficacy and Broad-Spectrum Antimicrobial Activity of pH-Controlled Sodium Hypochlorite Solution (HACCP’ER) in Acute and Chronic Wound Management: A Retrospective Cohort Study

**DOI:** 10.3390/jcm15114097

**Published:** 2026-05-26

**Authors:** Sadanori Akita, Toshihiko Okamura, Keisuke Tanigawa

**Affiliations:** 1 Department of Plastic and Reconstructive Surgery, Tamaki Aozora Hospital, Tokushima 779-3125, Japan; 2 Department of Bioregulation and Pharmacological Medicine, Fukushima Medical University, Fukushima 960-1295, Japan; 3 TechnoMax, LLC, Chiba 283-0822, Japan; siokamura@yahoo.co.jp; 4 Shinkou Co., Ltd., Nagasaki 867-0431, Japan; k.tanigawa@shinkou-inc.co.jp

**Keywords:** hypochlorous acid, pH-controlled sodium hypochlorite, wound healing, wound infection, antimicrobial, HACCP’ER, pressure ulcer, diabetic foot ulcer, burn, MRSA

## Abstract

**Background/Objectives:** Effective wound antisepsis and infection control remain central challenges in both acute and chronic wound management. pH-controlled sodium hypochlorite solution (HACCP’ER^®^) is a novel agent that optimizes the proportion of bactericidal hypochlorous acid (HOCl) by maintaining pH at 6.0–7.3. The present preliminary study aimed to evaluate its broad-spectrum antimicrobial activity in vitro and clinical outcomes in a retrospective cohort of patients with diverse acute and chronic wounds. **Methods:** A retrospective observational study was conducted, involving 193 consecutive patients who received HACCP’ER-based wound care between May 2022 and February 2023. Wound categories included pressure ulcers (n = 61), foot ulcers (n = 44), burns (n = 42), acute traumatic wounds (n = 29), and other chronic wounds (n = 17). HACCP’ER was applied at a free available chlorine (FAC) concentration of 50–200 ppm at pH = 6.0–7.3. In vitro antimicrobial suspension testing against ten microbial species was performed at 57 ppm (pH = 5.2, 23 °C) according to Japanese Industrial Standards. **Results:** HACCP’ER at 57 ppm eliminated *Escherichia coli*, *Staphylococcus aureus*, methicillin-resistant *S. aureus* (MRSA), *Streptococcus* spp., *Salmonella* spp., and *Pseudomonas aeruginosa* to below the detection limit (<10 CFU/mL) within 1 min, *Candida* within 3 min, and black *Aspergillus* within 5 min. In clinical wound cultures, bacterial burden was reduced in 6 of 10 (60%) patients. The mean patient age was 67.4 years. No adverse events attributable to HACCP’ER were recorded. Progressive wound healing was documented across all wound categories, with representative cases achieving closure at 1–11 months. **Conclusions:** HACCP’ER demonstrates potent broad-spectrum antimicrobial activity at wound-relevant concentrations and is clinically safe in acute and chronic wound care. Its physiologically aligned mechanism of HOCl generation supports both efficacy and biocompatibility. Prospective randomized controlled trials are warranted to definitively establish clinical efficacy.

## 1. Introduction

Wound infection and impaired healing represent persistent clinical challenges associated with significant morbidity, prolonged hospitalization, and high healthcare costs, with annual Medicare expenditures for wound care in the United States estimated at $28–$97 billion [1,2]. An ideal wound antiseptic must combine broad-spectrum antimicrobial activity with minimal cytotoxicity to host cells, ease of use, and low cost [3].

Sodium hypochlorite solutions have been employed in wound care since Dakin’s landmark work in the early twentieth century. However, conventional preparations at pH 8.5–10.0 usually contain the hypochlorite ion (OCl^−^), which is substantially less bactericidal and more cytotoxic to fibroblasts and keratinocytes than hypochlorous acid (HOCl) [4,5,6,7]. The antimicrobial potency of free available chlorine (FAC) is highly pH-dependent: at pH 5–7, HOCl constitutes more than 80% of total FAC, while at pH > 8, the less active OCl^−^ ion predominates. HOCl has been estimated to be approximately 80-fold more potent as a microbicide than OCl^−^ at equivalent FAC concentrations [8,9].

HACCP’ER^®^ (Shinkou Co., Ltd., Sasebo, Japan) is a novel pH-controlled sodium hypochlorite solution produced by a non-electrolytic dilution-mixing process (NaOCl + HCl + H_2_O → HOCl + NaCl + H_2_O) that maintains FAC predominantly as HOCl at pH 6.0–7.3. Unlike electrolyzed oxidizing water, HACCP’ER does not require dedicated on-site electrolysis equipment and allows flexible adjustment of FAC concentration (0.05–2000 ppm), pH (2.5–9.0), and temperature (4–85 °C) [10].

Mechanistically, exogenous HOCl mimics the endogenous myeloperoxidase (MPO)-catalyzed generation of HOCl in activated neutrophils (H_2_O_2_ + Cl^−^ → HOCl + OH^−^), which is a central component of innate antimicrobial immunity [11,12]. HOCl disrupts bacterial cell membranes by oxidizing sulfhydryl groups and selectively binding unsaturated lipid layers, leading to rapid bacteriolysis [13]. This physiological parallel supports the biocompatibility of pH-optimized HOCl formulations at wound-appropriate concentrations.

Despite growing clinical interest in HOCl-based products, rigorous data on pH-optimized sodium hypochlorite solutions across heterogeneous wound populations remain limited. The present study reports both in vitro antimicrobial efficacy of HACCP’ER against ten medically relevant microorganisms and a retrospective clinical series from a single center, aiming to characterize the safety and clinical utility of this agent across diverse wound categories.

## 2. Materials and Methods

### 2.1. Study Design and Ethical Considerations

This retrospective observational study included 193 consecutive patients treated at the wound management service of a university-affiliated hospital between May 2022 and February 2023. All patients provided written informed consent prior to enrollment. The study was conducted in accordance with the Declaration of Helsinki and was approved by the Institutional Review Board (approval no.: J-20022003). Patient data were anonymized for analysis. The study follows the STROBE guidelines for observational studies.

### 2.2. HACCP’ER^®^ Solution

HACCP’ER is manufactured by mixing 12% sodium hypochlorite (NaOCl, pH 12) and 8.5% hydrochloric acid (HCl, pH < 1) with reverse osmosis (RO) water in a dedicated mixing tank, yielding a stable HOCl-predominant solution at pH 6.0–7.3. At the target pH range, HOCl constitutes >75% of FAC. For clinical wound care applications, FAC concentrations of 50–200 ppm (mg/L) were used; for in vitro testing, a concentration of 57 ppm at pH 5.2 and 23 °C was employed. Delivery methods included jet irrigation, impregnated dressings, and vacuum-assisted contact.

### 2.3. Patient Population and Wound Classification

Wounds were categorized into five groups: (1) pressure ulcers (PU), including sacral, trochanteric, ischial, and heel ulcers; (2) foot ulcers (FU), including diabetic foot ulcers (DFU), peripheral arterial disease-related ulcers (PAD), and venous leg ulcers (VLU); (3) burns, including superficial and deep partial-thickness burns; (4) acute traumatic wounds, including lacerations, degloving injuries, and bite wounds; and (5) other chronic wounds, including Fournier’s gangrene, lymphedema ulcers, and radiation-induced wounds.

### 2.4. Wound Treatment Protocol

Standard wound care was applied at each visit or daily for inpatients, consisting of (1) debridement of necrotic and sloughy tissue; (2) wound irrigation with HACCP’ER (delivered as 100–200 mL per session through a 19-gauge blunt-tip cannula attached to a 60-mL syringe at a hand-controlled flow rate of approximately 10–15 psi, with a wound-bed contact time of 30–60 s, followed by application of gauze impregnated with HACCP’ER for an additional 5 min); and (3) appropriate wound dressings (polyurethane foam, hydrocolloid, antimicrobial dressings, or negative pressure wound therapy as indicated). Outcome data were extracted from medical records. Wound healing was prospectively assessed at every clinical visit (every 2–3 days for outpatients and daily for inpatients) using three pre-specified objective measures: (i) standardized digital photography from a fixed distance under uniform lighting with a calibrated in-frame ruler; (ii) planimetric wound surface area measurement (cm^2^) using ImageJ1.54 software (NIH, Bethesda, MD, USA) with the in-frame ruler as scale; and (iii) the Pressure Ulcer Scale for Healing (PUSH) tool for pressure ulcers and foot ulcers (range 0–17, lower is better) or the Bates–Jensen Wound Assessment Tool (BWAT, range 13–65, lower is better) for chronic wounds of other etiologies. Complete wound closure was defined as 100% re-epithelialization sustained for at least 14 days and was confirmed by two independent assessors (a board-certified wound nurse and a plastic surgeon). Percent wound area reduction was calculated at 4, 8, and 12 weeks as [(baseline area − current area)/baseline area] × 100.

### 2.5. Microbiological Evaluation

In a subset of 10 patients with chronic wounds clinically suspected of bacterial colonization, wound swab cultures were obtained pre- and post-treatment using a within-subject crossover design. Each patient first received a 1-week treatment course with benzalkonium chloride 0.05% (BAC; the institutional standard antiseptic) with pre- and post-treatment swabs, followed by a 1-week saline-only washout, then a 1-week course of HACCP’ER (FAC 100 ppm, pH 6.5) with a new set of pre- and post-treatment swabs. The same anatomical area of each wound was sampled at every time point by the Levine technique (rotating swab over a 1-cm^2^ area with sufficient pressure to express tissue fluid). For four patients (denoted N/T in Table 1, data were available only for the HACCP’ER arm because these patients entered the wound clinic after the BAC arm had been completed elsewhere. Cultures were processed by the central hospital microbiology laboratory using standard aerobic and anaerobic methods on blood, MacConkey, and Sabouraud agar. Semi-quantitative culture grading was used: negative (−), sparse (+), moderate (++), or heavy (+++).

### 2.6. In Vitro Antimicrobial Suspension Testing

In vitro bactericidal/fungicidal testing was performed according to the Japanese Industrial Standards (JIS) Z 2801 suspension method. Microbial suspensions at 10^5^–10^6^ CFU/mL were prepared and exposed to three test agents for 1, 3, and 5 min at 23 °C: (1) HACCP’ER at 57 ppm, pH 5.2; (2) benzalkonium chloride solution (positive control); and (3) commercial sodium hypochlorite at 200 ppm, pH 8.4. Organisms tested included *E. coli*, *S. aureus*, MRSA, *Streptococcus* spp., *Salmonella* spp., *P. aeruginosa*, *Bacillus subtilis* (spore-forming), *Candida* spp., and black *Aspergillus*. The detection limit was <10 CFU/mL.

### 2.7. Statistical Analysis

Descriptive statistics (mean ± standard deviation for normally distributed continuous variables; median and interquartile range for non-normally distributed variables; counts and percentages for categorical variables) were calculated. Comparison of pre- versus post-treatment culture grades within the same patient used the Wilcoxon signed-rank test; between-arm comparison of binary culture-negativity outcomes used Fisher’s exact test. Wound area reduction at 4 weeks was compared across the five wound categories by Kruskal–Wallis test, with post hoc Dunn’s test where appropriate. PUSH and BWAT score changes over time were analyzed by repeated-measures non-parametric tests. Inter-rater agreement for wound closure determination was assessed by Cohen’s kappa. Statistical significance was defined as two-tailed *p* < 0.05. Analyses were performed using SPSS version 28.0 (IBM, Armonk, NY, USA), and descriptive tables were cross-checked in Microsoft Excel (version 2019). Given the exploratory and preliminary nature of this study, no formal adjustment for multiple comparisons was applied, and reported *p*-values should be interpreted as hypothesis-generating.

## 3. Results

### 3.1. Patient Demographics and Wound Distribution

A total of 193 patients (mean age 67.4 years; range 4–101 years) were enrolled. Table 2 summarizes the distribution by wound category and mean age decade.

Pressure ulcers were the most prevalent category (31.6%), occurring predominantly in elderly patients (mean age decade 8.3). Sacral pressure ulcers constituted the most frequent subtype, with 13 Stage 4 sacral ulcers documented. Foot ulcers (22.8%) included ischemic, diabetic, and venous etiologies. Burns (21.8%) ranged from superficial to deep partial-thickness injury. Acute traumatic wounds (15.0%) included lacerations (n = 12), degloving injuries (n = 4), and animal bite wounds (n = 3).

### 3.2. In Vitro Antimicrobial Efficacy

HACCP’ER at 57 ppm (pH 5.2, 23 °C) achieved complete elimination (<10 CFU/mL) of all six vegetative bacterial species within 1 min. *Candida* spp. were eliminated within 3 min and black *Aspergillus* within 5 min. *B. subtilis* spores showed partial resistance at 1 min (3.7 × 10^5^ CFU/mL) but complete elimination at 3 min. Full results are presented in Table 3.

### 3.3. Clinical Wound Culture Results

Wound swab cultures were obtained from 10 patients pre- and post-treatment. The HACCP’ER group showed bacterial burden reduction in 6 of 10 patients (60%, 95% CI 31–83%) and complete culture negativity in 3 of 10 patients (30%, 95% CI 11–60%). In the BAC comparator group, reduction was observed in 3 of 10 patients (30%, 95% CI 11–60%). Between-arm comparison of any reduction did not reach statistical significance (Fisher’s exact test, *p* = 0.37); the Wilcoxon signed-rank test on ordinal pre-/post-treatment culture grade change likewise yielded *p* = 0.18. These exploratory results are consistent with the small sample size and the hypothesis-generating nature of this comparison. Table 1 presents individual results.

### 3.4. Clinical Wound Healing Outcomes

Among the 193 enrolled patients, 178 (92.2%) completed at least one follow-up visit at 4 weeks and were included in the wound-healing analysis. The mean percent wound area reduction at 4 weeks varied across categories: acute traumatic wounds 71 ± 15%, burns 62 ± 18%, pressure ulcers 44 ± 21%, foot ulcers 32 ± 26%, and other chronic wounds 30 ± 22% (Table 4). Venous leg ulcers showed the slowest response, consistent with their well-recognized indolent natural history. Complete wound closure within the median follow-up of 5.8 months was achieved in 38/42 burns (90.5%), 24/29 acute traumatic wounds (82.8%), 39/61 pressure ulcers (63.9%), 21/44 foot ulcers (47.7%), and 8/17 other chronic wounds (47.1%). In the pressure ulcer subgroup, PUSH scores decreased from a baseline mean of 13.2 ± 2.4 to 6.8 ± 4.1 at 12 weeks (Wilcoxon signed-rank *p* < 0.001). Inter-rater agreement for closure determination was substantial (Cohen’s κ = 0.81). Because of the uncontrolled retrospective design, these healing outcomes should not be interpreted as direct evidence of efficacy of HACCP’ER in isolation; they reflect outcomes from a multi-component wound care protocol in which HACCP’ER was one element.

### 3.5. Representative Clinical Cases

**Figure 1:**
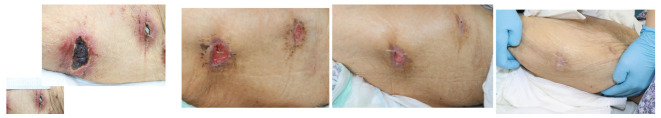
Case 1. Left trochanteric Stage 4 pressure ulcer in an 85-year-old female. From left to right: presentation, 1 month, 2 months, and 5 months after initiation of HACCP’ER irrigation. Note progressive resolution of necrotic eschar, granulation tissue formation, and near-complete re-epithelialization by month 5.

**Case 1 (Pressure Ulcer):** An 85-year-old female with a left trochanteric Stage 4 pressure ulcer was treated with daily HACCP’ER irrigation. Progressive granulation tissue formation was observed from month 1, with near-complete re-epithelialization at month 5 (Figure 1).

**Figure 2:**
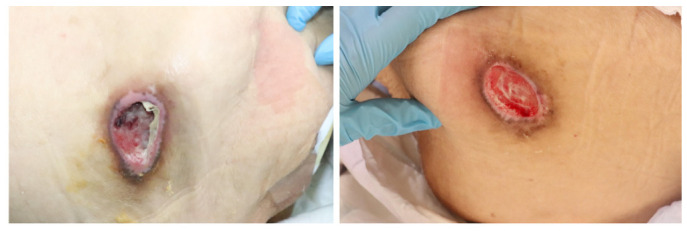
Case 2. Right trochanteric Stage 4 pressure ulcer in a 75-year-old male. Left: presentation showing slough and necrotic tissue. Right: 2 months after HACCP’ER application showing healthy granulation and contraction.

**Case 2 (Pressure Ulcer):** A 75-year-old male with bilateral Stage 4 trochanteric pressure ulcers showed marked reduction in sloughing and wound surface area at 2 months (Figure 2).

**Figure 3:**
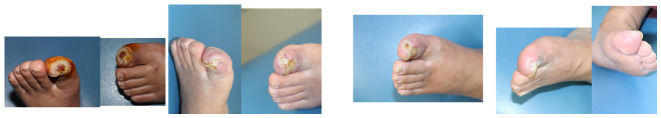
Case 3. Recurrent left first-toe diabetic foot ulcer in a 54-year-old male. Time course (left to right): presentation, 1 month, 2 months, and 3 months. Complete re-epithelialization was achieved at 3 months.

**Case 3 (Diabetic Foot Ulcer):** A 54-year-old male with a recurrent left first toe DFU with bone involvement achieved complete re-epithelialization at 3 months following HACCP’ER irrigation combined with surgical debridement (Figure 3).

**Figure 4:**
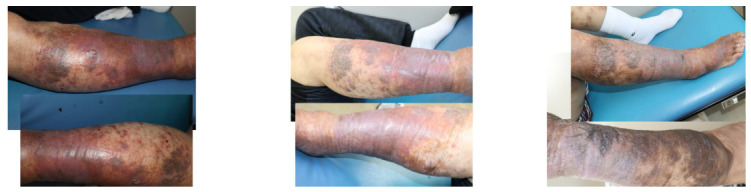
Case 4. Bilateral lower-extremity venous leg ulcers in a 66-year-old male. (**Left**): presentation with multiple ulcerations and surrounding hyperpigmentation. (**Center**): at 1 month showing partial closure. (**Right**): at 11 months showing sustained healing with residual hemosiderin staining.

**Case 4 (Venous Leg Ulcer):** A 66-year-old male with bilateral VLU demonstrated sustained wound healing over 11 months of outpatient management (Figure 4).

**Figure 5:**
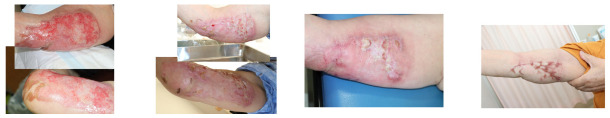
Case 5. Right arm Grade II partial-thickness burns covering 4% of total body surface area in an 80-year-old female. From left to right: presentation, 2 weeks, 4 weeks, and 7 months. Complete spontaneous re-epithelialization was achieved without surgical grafting.

**Case 5 (Burns):** An 80-year-old female with right arm superficial-to-deep partial-thickness burns covering 4% TBSA achieved complete healing by month 7 without skin grafting (Figure 5).

No systemic adverse events, contact dermatitis, chemical burns, or hypersensitivity reactions attributable to HACCP’ER were observed in any patient during the study period.

## 4. Discussion

The present study demonstrates that pH-controlled sodium hypochlorite solution (HACCP’ER) exhibits broad-spectrum, rapid antimicrobial activity in vitro and was used safely—without observed adverse effects—in a retrospective clinical cohort in which progressive wound healing was documented across diverse wound categories. We emphasize that the uncontrolled retrospective design precludes any causal attribution of clinical healing to HACCP’ER specifically. These findings are consistent with the growing body of literature supporting HOCl-based antiseptics as effective and biocompatible wound care agents [14,15].

The critical innovation of HACCP’ER lies in pH optimization. The chlorine speciation equilibrium is strongly pH-dependent: below pH 7.5, HOCl constitutes the dominant chlorine species and is approximately 80-fold more potent as a microbicide than OCl^−^ [8,9]. Commercial sodium hypochlorite preparations at pH 8.5–9.5 exist almost entirely as OCl^−^, with substantially reduced bactericidal activity. HACCP’ER, by maintaining pH at 6.0–7.3, delivers a solution in which >75% of FAC is the active HOCl species.

HACCP’ER can be conceptually compared with three categories of clinically available wound cleansers. First, traditional Dakin’s solution (0.5% NaOCl, pH 9–10) remains in use but is increasingly viewed as cytotoxic at active concentrations [3,7]. Second, electrolyzed-water-based stabilized HOCl products (Microcyn^®^, Dermacyn^®^, Vashe^®^) maintain HOCl as the dominant species but typically at lower FAC concentrations (50–200 ppm) and require shelf-life-managed pre-packaged formats [16]. Recent expert recommendations identify stabilized HOCl as a useful first-line antiseptic for infected wounds [16], and clinical reports describe favorable post-procedure healing with HOCl gels [17]. Third, alternative non-chlorine antiseptics such as polyhexanide (PHMB) and octenidine are increasingly preferred in European wound centers [18]; a 2023 systematic review and meta-analysis of polyhexanide versus HOCl-based antiseptics in chronic wounds reported comparable healing rates with no statistically significant difference, supporting HOCl-class agents as a cost-effective option [19]. HACCP’ER’s in-line preparation approach permits on-demand FAC and pH adjustment, which may offer practical advantages over pre-packaged HOCl in heterogeneous clinical settings, but this requires confirmation in adequately powered head-to-head trials.

The in vitro data confirm the potency of HACCP’ER against all clinically relevant gram-positive and gram-negative pathogens tested. The microbial species selected for testing reflect the pathogen spectrum consistently identified in metagenomics studies of chronic wound microbiota [20]. The rapid kinetics (complete kill within 1 min at 57 ppm) are comparable to findings by Herruzo et al. [14]. Notably, HACCP’ER achieved complete elimination of *B. subtilis* spores at 3 min, a performance profile superior to both BAC and commercial sodium hypochlorite at 200 ppm (pH 8.4), consistent with the well-established pH-dependence of chlorine sporicidal activity [9,21,22]. Benzalkonium and related quaternary ammonium antiseptics have been shown to impair wound healing in animal models [23], further supporting the clinical rationale for HOCl-based antiseptics.

The cytotoxic potential of conventional topical antiseptics was established by Lineaweaver et al. [5], corroborated by Cooper et al. [6]. Sodium hypochlorite-induced cytotoxicity has been attributed to ATP depletion and inhibition of DNA synthesis, most pronounced under alkaline conditions [7]. Heggers et al. identified 0.025% (250 ppm) as the threshold below which bactericidal activity is preserved without fibroblast toxicity [24]. The clinical HACCP’ER concentrations (50–200 ppm) are well within this nontoxic range [24,25], and the absence of observed adverse events in 193 patients supports this assessment.

Several important limitations must be explicitly acknowledged, and they should temper the interpretation of our findings. First, the retrospective uncontrolled observational design precludes causal attribution of clinical wound healing to HACCP’ER; the wound improvements we observed could plausibly be explained by concurrent debridement, dressing optimization, infection management, or the natural history of healing in the absence of any antiseptic agent. The proper experimental control—a parallel matched cohort receiving an alternative antiseptic with otherwise identical care—was not available. Second, the microbiological subset (n = 10) is too small to permit statistically meaningful between-treatment inference; the Fisher’s exact test (*p* = 0.37) is markedly underpowered and the apparent 60% vs. 30% difference may reflect chance variation. Third, wound area measurements, although prospectively recorded with planimetric software, were not blinded to treatment assignment, introducing potential measurement bias. Fourth, the study was conducted at a single center with one principal investigator, limiting external validity. Fifth, follow-up duration was heterogeneous (1–18 months), and survival/loss-to-follow-up analyses were not performed. Sixth, and importantly, two of the authors (T.O., K.T.) have financial relationships with the manufacturer of HACCP’ER—although the funders had no role in study design, data analysis, or manuscript preparation, this disclosed relationship represents a potential source of bias that readers should consider when weighing the conclusions. To mitigate this risk, outcome assessment was performed by hospital wound-care nursing staff and a board-certified plastic surgeon (S.A.) who is not employed by either commercial entity, but future trials should incorporate fully independent outcome adjudication, pre-registration on a public clinical-trial registry, and external statistical analysis. Seventh, the in vitro testing was performed in suspension format; biofilm-based assays would provide additional clinically relevant information, particularly for chronic wounds where biofilms predominate [20,26]. The findings of the present study should therefore be interpreted as preliminary and hypothesis-generating. Adequately powered, prospective, randomized, controlled trials comparing HACCP’ER with established antiseptics (such as polyhexanide, octenidine, or stabilized HOCl [16,19]) in well-defined wound categories—for example diabetic foot ulcers [27] or venous leg ulcers—with blinded outcome assessment, standardized planimetric wound measurement, quantitative cultures, and pre-specified primary endpoints, are required to establish the role of HACCP’ER in evidence-based wound care protocols.

## 5. Conclusions

pH-controlled sodium hypochlorite solution (HACCP’ER) at FAC concentrations of 50–200 ppm and pH 6.0–7.3 demonstrates broad-spectrum, rapid bactericidal and fungicidal activity in vitro, achieving complete eradication of common wound pathogens—including MRSA and *P. aeruginosa*—within 1 min at 57 ppm. In this preliminary retrospective series of 193 patients with diverse acute and chronic wounds, HACCP’ER was used as part of a multi-component wound care protocol; progressive wound healing was observed across all wound categories, and no safety signal attributable to HACCP’ER was identified. Because the study lacked a concurrent control arm, causal attribution of healing to HACCP’ER alone is not possible. The within-subject microbiological data (n = 10) suggested a numerical trend toward greater bacterial-burden reduction with HACCP’ER than with benzalkonium chloride, but the between-arm difference did not reach statistical significance. Because no mechanistic in vivo experiments were performed in the present study, the physiological rationale for the pH-controlled HOCl generation mechanism remains supported by prior research [8,9,11,12] rather than by direct evidence from our own data. These preliminary findings—explicitly framed as hypothesis-generating—support further evaluation of HACCP’ER as a candidate antiseptic in wound management, with adequately powered, blinded, prospective trials as the necessary next step.

## Figures and Tables

**Table 1:** Semi-quantitative wound swab culture results (pre- and post-treatment). − = culture-negative; + = sparse; ++ = moderate; +++ = heavy growth. BAC = benzalkonium chloride; N/T = not tested. † Culture-negative at post-treatment assessment.

Patient	Organism	BAC Pre	BAC Post	HACCP’ER Pre	HACCP’ER Post
1	*E. coli*	+	+	−	−
2	*E. coli*	++	++	++	++
3	*E. coli*	+	+	+	+
2	*S. aureus*	++	+	+	− †
4	*S. aureus*	N/T	N/T	+	− †
3	MRSA	+++	++	++	− †
5	MRSA	N/T	N/T	++	+
6	MRSA	N/T	N/T	+	+
7–10	*P. aeruginosa*	variable	unchanged	+	− †

**Table 2:** Patient distribution by wound category. * Age decade: 1 = 1–10 years through 10 = ≥91 years. DFU = diabetic foot ulcer; PAD = peripheral arterial disease; VLU = venous leg ulcer.

Wound Category	n	% of Total	Mean Age Decade *	Typical Age Range (Years)
Pressure Ulcer	61	31.6%	8.3	81–90
Foot Ulcer (DFU/PAD/VLU)	44	22.8%	8.1	81–90
Burns	42	21.8%	4.2	31–40
Acute Traumatic Wound	29	15.0%	5.3	41–50
Other Chronic Wound	17	8.8%	6.8	61–70
**Total**	**193**	**100%**	**6.8**	**4–101**

**Table 3:** In vitro antimicrobial suspension test results. HACCP’ER: 57 ppm, pH 5.2, 23 °C; BAC: benzalkonium chloride; NaOCl: sodium hypochlorite (200 ppm, pH 8.4, 23 °C); N/T: not tested at this time point. † Partial reduction at 1 min, complete elimination at 3 min. Detection limit: <10 CFU/mL.

Organism	Initial (CFU/mL)	HACCP’ER 1 min	HACCP’ER 3 min	HACCP’ER 5 min	BAC 1 min	NaOCl 200 ppm 1 min
*E. coli*	4.3 × 10^6^	<10	<10	<10	<10	<10
*S. aureus*	4.5 × 10^6^	<10	<10	<10	<10	<10
MRSA	3.4 × 10^6^	<10	<10	<10	<10	<10
*Streptococcus*	1.9 × 10^6^	<10	<10	<10	<10	<10
*Salmonella*	3.4 × 10^5^	<10	<10	<10	<10	<10
*P. aeruginosa*	1.6 × 10^5^	<10	<10	<10	<10	<10
*Candida*	2.3 × 10^6^	2.5 × 10^3^	<10	<10	2.5 × 10^3^	N/T
Black *Aspergillus*	2.0 × 10^5^	2.0 × 10^2^	30	<10	>10^4^	N/T
*B. subtilis* (spores)	4.6 × 10^6^	3.7 × 10^5^ †	<10	<10	>4.3 × 10^6^	>4.2 × 10^6^

**Table 4:** Clinical wound-healing outcomes by category. Area reduction is given as mean ± SD. Complete closure assessed within the median follow-up of 5.8 months.

Category	n Analyzed	Area Reduction at 4 wk (%)	Area Reduction at 12 wk (%)	Complete Closure n (%)
Pressure Ulcer	56	44 ± 21	68 ± 24	39/61 (63.9%)
Foot Ulcer	41	32 ± 26	54 ± 28	21/44 (47.7%)
Burns	40	62 ± 18	94 ± 8	38/42 (90.5%)
Acute Traumatic	27	71 ± 15	96 ± 6	24/29 (82.8%)
Other Chronic	14	30 ± 22	52 ± 30	8/17 (47.1%)

## Data Availability

The data presented in this study are available on request from the corresponding author. The data are not publicly available due to patient privacy regulations.
